# Cross-leg flap: Its role in limb salvage

**DOI:** 10.4103/0019-5413.43394

**Published:** 2008

**Authors:** Pawan Agarwal, HKT Raza

**Affiliations:** Plastic Surgery Unit, Department of Surgery and Orthopedics, N.S.C.B. Government Medical College, Jabalpur, India

**Keywords:** Cross-leg flap, free tissue transfer, lower limb trauma

## Abstract

**Background:**

Pedicled cross-extremity flaps for lower limb wound coverage have been replaced by free tissue transfer in the last two decades. However, there are certain difficult situations where the free flap cannot be employed and alternative methods are needed. We describe our experience with cross-leg flap in 18 patients for the reconstruction of difficult leg defects in which no suitable recipient vessels were available for microvascular anastomosis in the vicinity of the defect.

**Materials and Methods:**

18 patients (17 men and 1 woman) with mean range 31.5 yrs(range 18-70 yrs) grade III B tibial fractures were included in the study. fasciocuteneous cross leg flap was employed and extremities were immobilized by external Fixator.

**Results:**

Fifteen flaps were completely available with two had marginal necrosis and one supsficial epidermal necrosis. No complications were related to the donor site, flap, or by immobilization are noted. Each patient resumed essentially normal gait and activity without any stiffness of joints related with the flap or external fixator.

**Conclusion:**

The addition of external fixator stabilization aids greatly in wound care, as well as for general ease of the patient mobility and positioning. Cross-leg flap offers the possibility of salvaging limbs that are otherwise nonreconstructable.

## INTRODUCTION

Cutaneous injuries of the lower third of the leg and dorsum of the foot represent a great challenge for orthopedic and plastic surgeons. The poor vascularization and subsequent poor healing encountered in these regions demand detailed knowledge of the local anatomy to select the best surgical alternative for each patient. The free flaps are usually the first choice for soft tissue coverage in the distal leg. There continue to be, however, some clinical situations in which local fasciocutaneous and myocutaneous flaps are often not available. Occasionally, a free flap may also have failed because of technical errors or damaged vasculature. In these situations, a cross-leg flap is the best choice. The inclusion of fascia in the flap makes length-to-breadth ratio 3: 1 perfectly safe. This allows much greater area of skin to be transferred with much more freedom of leg position.^1^,^2^ The flap provided stable coverage for different defects with few complications.^3^ Even should the flap fail, no significant bridges have been burnt and all the other surgical options remain viable. Traditionally, cross-leg flaps have been problematic because of difficulties with immobilization and positioning of the extremities from the time of initial coverage to detachment. The use of external fixator for immobilization circumvents many of these problems and facilitates the use of cross-leg flaps in patients in whom free tissue transfer may not be optimal.^4^

## MATERIAL AND METHODS

**Figure 1:**
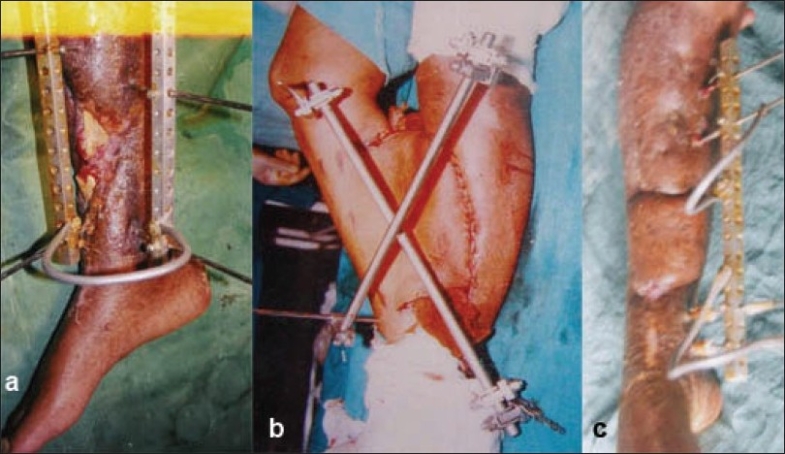
(a) Clinical photograph shows compound middle one-third both bones leg fracture and on exploration no suitable vessel was available. (b) Clinical photograph shows cross leg flap and fixation of two legs with external fixator. (c) Clinical postoperative photograph showing cross-leg flap after division.

The records of all patients who had cross-leg flap for trauma in the last 2½ years were reviewed. During this period, 18 patients (17 men and 1 woman) with mean age of 31.5 years (range 18–70 years) were seen. High-velocity road traffic accident was the predominant cause and occurred in 17 patients; the remaining one patient had high-voltage electrical burn in the lower limb. All the posttrauma patients had Type IIIB tibial fractures, and lower one-third of the leg was the most common site (n = 8); middle one-third defect (n = 7) and two-third length of lower limb was involved in three patients. In three patients, the soft tissue loss was circumferential; in three patients, it extended beyond half of the circumference, and in the remaining, it involved the half of the circumference of the leg. There was associated fracture of fibula in 10 cases. Anterior tibial and peroneal vessel injury was seen in four cases, which was detected by color Doppler study. Associated injury to other sites was found in four patients. In all the cases, fasciocutaneous cross-leg flap was employed, and extremities were immobilized by external fixator [Figure 1 a–b].

### Surgical procedure

The calf was the principle donor site for cross-leg flap. Precise preoperative planning was done, and the proposed defect was outlined including scarred local tissue. The flap was planned using a cloth pattern of the defect with generous margin and to include the length of bridge as short as possible. Keeping the limb in a comfortable position avoiding extreme joint flexion, planning in reverse was done. Precaution was taken to avoid any twisting or kinking of flap in the final position of the limb. The flap was based preferably proximally or anteromedially depending upon the location of the defect, with a proportion of up to 3: 1. Transverse flap based anteriorly on the medial calf was based at least 3 cm behind the medial border of the tibia to preserve the long sephanous vein and rows of important perforators. The defect was debrided, and the margin was freshened. After proper preoperative marking, the flap was raised including the fascia. The donor site of the flap was split skin grafted, and the flap was sutured over the defect. Both the limbs were kept in position using the external fixator. In 10 patients, the traditional anteromedial based flap was used; in six patients, superiorly based flap was used; and in remaining two patients, inferiorly based flap was used. The choice of location of the base of the flap depends on the comfortable position of the leg.

Division of the flap was performed in patients after mean 21 days (range 15–24 days), and preliminary delay was performed in three patients. The mean follow-up period was 18 months. At follow-up, the patients were evaluated for functional as well as for cosmetic outcome.

## RESULTS

**Figure 2:**
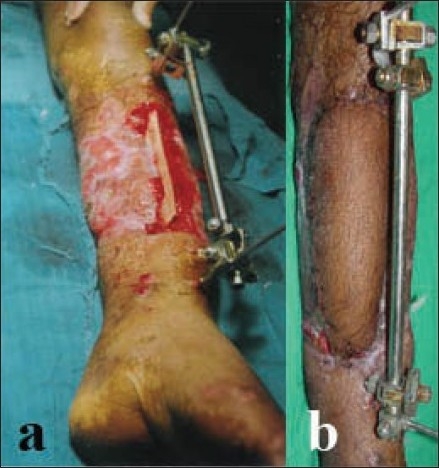
(a) Clinical photograph shows circumferential wound with fracture middle one- third both bone legs. (b) First stage skin grafting was done and later cross-leg flap covered the exposed fracture site.

**Figure 3:**
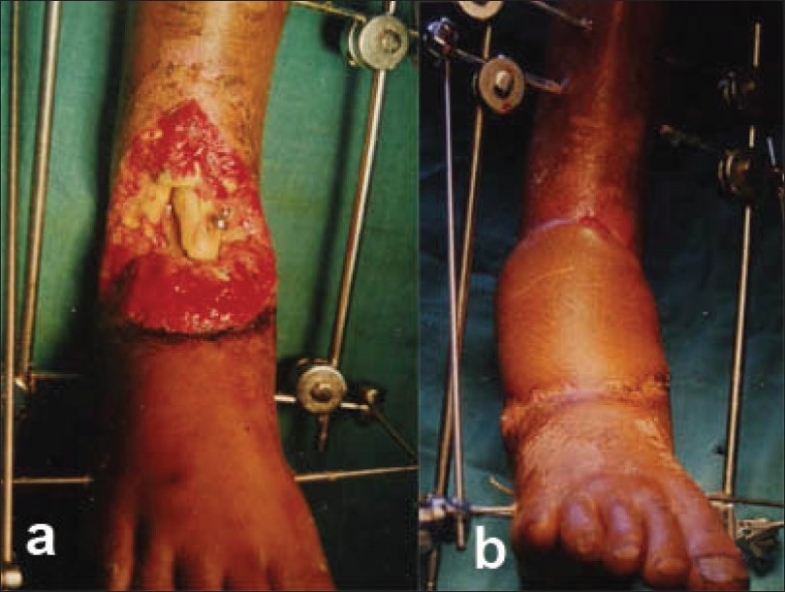
(a) Clinical photograph shows compound fracture lower end both bones leg involving the ankle mortise. (b) Postoperative cross-leg flap in place.

Fifteen flaps were completely viable after division. Two patients had marginal necrosis and one patient had superficial epidermal necrosis, all healed completely in one week. Partial graft loss on the flap donor site was seen in one patient, which required regrafting. One patient who had associated fracture shaft femur had restriction of knee movement. Another patient who had fractured lower one-third of tibia and fibula involving the ankle mortise had stiff ankle joint. From the plastic surgical point of view, all the flaps served the reconstructive purposes, and none of them required major secondary procedure, but 10 patients required secondary orthopedic procedures such as bone grafting or Ilizarov for fracture healing. Other relevant details of our 18 patients and the methods employed are shown in Table 1 and Figures 1–3.

**Table 1:** Details of patients and the methods employed

Case number	Age (years)	Sex	Location of defect	Flap size (cm)	Days of flap coverage	Secondary procedures	Results	Follow-up (weeks)
1	24	M	Recipient vessel not available middle one-third [Figure 1b]	16 × 8	35	Bone graft	Complete survival [Figure 1c]	24
2	28	M	Failed previous free flap lower one-third with segmental loss of TF	15 × 7	30	Ilizarove	Complete survival	Under follow-up
3	22	M	Electrical burns lower one-third, major venous thrombosis	12 × 8	14	Nil	Complete survival	4
4	26	M	Upper two-third major lower extremity injury with axial vessels damage	14 × 7	21	Bone graft	Complete survival	Under follow-up
5	30	M	Lower one-third delayed referral,	17 × 8	58	Nil	Marginal necrosis	24
6	70	F	Upper two-third delayed referral single vessel limb	20 × 10	60	Bone graft	Marginal necrosis	36
7	20	M	Middle one-third circumferential injury with single vessel limb [Figure 1c]	16 × 9	54	Bone graft	Complete survival [Figure 4]	32
8	24	M	Middle one-third delayed referral, severely scarred compromised limb,	18 × 8	50	Nil	Complete survival	36
9	45	M	Lower one-third delayed referral with diabetes mellitus [Figure 2a]	17 × 8	45	Nil	Complete survival [Figure 2b]	22
10	28	M	Lower one-third delayed referral,	16 × 8	52	Nil	Complete survival	24
11	60	M	Lower two-third Circumferential injury, Delayed referral	18 × 10	48	Nil	Complete survival	20
12	40	M	Lower one-third delayed referral	20 × 9	30	Bone graft	Complete survival	29
13	36	M	Middle one-third, single vessel limb	20 × 9	6	Ilizarove	Complete survival	26
14	25	M	Middle one-third delayed referral	21 × 13	41	Ilizarove+Bone graft	Complete survival	24
15	18	M	Lower one-third recipient vessel not available	18 × 8	6	Nil	Complete survival	28
16	22	M	Middle one-third recipient vessel not available	20 × 9	2	Nil	Complete survival	26
17	25	M	Middle one-third Circumferential injury	22 × 10	1	Bone graft	Complete survival	22
18	24	M	Lower one-third, severely scarred compromised limb	16 × 7	45	Bone graft	Complete survival	32

## DISCUSSION

An injury to the lower extremity can be a complex problem, often involving the fractured bone, exposed tendons, and soft tissue defects. Microsurgical free flap is now a well-established procedure in the reconstruction of severely damaged lower extremities. However, successful result depends on the availability of suitable vessel with healthy vascular wall and adequate size for microvascular anastomosis. Hamilton first introduced the cross-leg flap in 1854. During the Second World War, this flap was used extensively with gratifying results. Stark(1950) standardized the procedure and summarized its usefulness for lower extremities trauma.^5^ Since the introduction of free flap in (1970), it has become the gold standard for lower extremity reconstruction, and indications for cross-leg flap declined. However, the situation occasionally arises in which an alternative method may be necessary and cross-leg flap becomes a simple and effective option that should remain in the armamentarium of plastic surgeons.

Free flaps cannot be used in patients with major lower extremity injury with axial vessel damage and a history of previous trauma and thrombosis of vessels. Failed previous free flap presents special problems in reconstruction. Locally diseased arterial tree, recipient vessel not available on exploration, and general condition of the patient not permitting long-standing surgery forms other contraindications for free flap. Relative contraindications of free flap include electrical burns, single vessel limb, delayed referral, and in patients after bone tumor resection that had radiotherapy. In pediatric age group, it is fraught with technical difficulties. In these situations, the cross-leg fasciocutaneous flap can be a good alternative to reconstruct the defects. The indications may be markedly broadened especially in the centers with no access to microsurgery. Hence, the cross-leg flap becomes a valuable option in the aforementioned conditions.

With the advent of microsurgery, increased emphasis has been placed on the use of free tissue transfer for coverage of extensive lower extremity defects. Even in the aforementioned conditions, the use of cross-leg flap can be debated, and some surgeons have used other techniques like prefabrication of vascular pedicle and “carrier” vessels of the contralateral noninjured leg to perfuse the flap.^6^–^9^ Again these procedures required microvascular expertise and are carried out in two stages, therefore loosing the advantages of microsurgical single-stage procedure. Significant donor site deformity and morbidity, long operative hours, and secondary revision for debulking and contouring of the flap specially used around heel and ankle may be considered as additional disadvantages with free tissue transfer.

In cases of delayed referral due to head, chest, or abdominal trauma, which gets priority over limb injury, the granulation tissue and extensive fibrosis around the bony defect and perivascular fibrosis extending several centimeters away from the visible limit of trauma leads to the unavailability of recipient vessel near the defect. There is significant difference in the failure rate between those patients having free flap less than three days from the time of injury and those having flaps more than three days to three months after the injury.^10^ Once the free flap is failed, there is limited option available. After failed free flap, the rate of amputation of extremity varies from 22 to 57% in different studies.^11^–^13^ Despite failed free flap, majority of the extremities can be salvaged by split skin graft and local flaps, but intermittent wound breakdown and drainage of extremities remained major problems. This indicates that loss of a free flap significantly affects the potential to salvage a lower extremity.^14^ However, after previous failed free flap, if the same indication remains, a second free flap is not a contraindication, but it again carries a risk of failure. Patients with electrical burn, locally diseased arterial tree especially in cases of diabetes and Buerger's disease, and in patients who had radiotherapy will have higher complications in terms of arterial and venous thrombosis. In cases of single vessel limb end-to-side anastomosis can be performed but there is always a chance of vascular thrombosis with subsequent threat to limb viability.

The use of cross-leg flap has previously been limited by the incidence of necrosis, difficulty of immobilizing both legs for 2–3 weeks, joint stiffness, chances of thromboembolism, and concern about donor site cosmetic deformity especially in women. The use of external fixator for immobilization circumvents many of the previous problems with both-leg immobilization. By incorporating fascia or muscle, the reliability of the flap can be enhanced, and flap can be raised in a proportion up to 3: 1 to 5: 1.^15^ Cosmetic deformity can be reduced by using fasciosubcutaneous flap. External fixator is quick and easy to apply, light in weight, less awkward both for the patient and for nursing personnel, and is easy to adjust in the ward. It provides the necessary strength for immobilization and overhead suspension. There were no complications related to the donor site or flap itself or caused by the fixation. Lower-extremity range of motion is regained rapidly, and each patient resumed essentially normal gait and activity. The addition of external-fixator stabilization aids greatly in wound care, as well as for general ease of patient mobility and positioning.^16^

In our study, the mean length of time from first operation to complete healing was 32 days, and the mean operating time for both the stages was 2½ h. Stable coverage was obtained in all the patients. Wells *et al*. reported that Type IIIB tibial fractures carried a significantly higher risk of free-flap failure than the other types of fracture, and stable, long-term coverage of the free flaps was achieved only in 78% of patients.^17^ For free flap coverage in lower limb, Serafin *et al*. reported the average time in the hospital as 36.2 days and average operating time as 8 h.^18^ Morris *et al*. reported 94% success rate with conventional cross-leg flap, and by incorporating the fascia, the success rate approaches nearly 100%.^19^ For free flap, most of the centers reports success rate in the range of 90–92%.^20^ However, recently many centers have reported considerable success with free flap with short operating time, but these are the best centers for microsurgery with considerable experience, and very few centers can simulate their results.

Cross-leg flaps remain a useful and highly reliable tool for the reconstruction of difficult wounds of the lower limb.^21^ It offers the possibility of salvaging limbs that are otherwise nonreconstructable. Cross-extremity flaps function as a nutrient flap for the distal limb even though the pedicle has been divided.^22^ It is a backup procedure in an urgent situation and supplies a large quantity of skin. Advantages of cross-leg flap include ease of dissection, versatility, shorter operating time, minimal donor site morbidity, and replacement of like tissue with little or no need for secondary revision.^23^

With its simplicity, reliability, absence of functional deficit, and good-quality coverage with only moderate aesthetic disadvantage, cross-leg flap finds a definite place in reconstructive trauma surgery.^24^ Therefore, we recommend its use for injuries unsuitable for local tissue transfer, when real microvascular expertise is not available or operating room time is restricted.

## CONCLUSION

The cross-leg flap is a safe and reliable alternative to free tissue transfer in certain situations of lower-limb trauma. By incorporating fascia or muscle and the use of external fixator, the versatility of the flap can be enhanced. This flap is easy to perform and does not require the sophisticated equipment or expertise of microanastomosis.

## References

[1] Calhoun JH, Gogan WJ, Beraja V, Howard RJ, Oliphant JR (1989). Dynamic axial fixation for immobilization of cross-leg flaps in chronic osteomyelitis. Ann Plast Surg.

[2] Barclay TL, Sharpe DT, Chisholm EM (1983). Cross-leg fasciocutaneous flaps. Plast Reconstr Surg.

[3] de Almeida OM, Monteiro AA, Neves RI, de Lemos RG, Braz JC, Brechtbuhl ER (2000). Distally based fasciocutaneous flap of the calf for cutaneous coverage of the lower leg and dorsum of the foot. Ann Plast Surg.

[4] Velazco A, Fleming LL, Nahai F (1983). Soft-tissue reconstruction of the leg associated with the use of the Hoffmann external fixator. J Trauma.

[5] Stark RB (1952). The cross leg flap procedure. Plast Reconstr Surg.

[6] Devansh S (1995). Prefabricated recipient vascular pedicle for free composite-tissue transfer in the chronic stage of severe leg trauma. Plast Reconstr Surg.

[7] Yamada A, Harii K, Ueda K, Asota H, Tanaka H (1995). Versatility of a cross leg free rectus abdominis flap for leg reconstruction under difficult and unfavorable conditions. Plast Reconstr Surg.

[8] Lai CS, Lin SD, Chou CK, Cheng YM (1991). Use of a cross-leg free muscle flap to reconstruct an extensive burn wound involving a lower extremity. Burns.

[9] Chen H, El-Gammal TA, Wei F, Chen H, Noordhoff MS, Tang Y (1997). Cross-leg free flaps for difficult cases of leg defects: Indications, pitfalls, and long-term results. Trauma.

[10] Godina M (1986). Early microsurgical reconstruction of complex trauma of the extremities. Plast Reconstr Surg.

[11] Weiland AJ, Moore JR, Daniel RK (1984). The efficacy of free tissue transfer in the treatment of osteomyelitis. J Bone Joint Surg Am.

[12] Swartz WM, Mears DC (1985). The role of free tissue transfer in lower extremities reconstruction. Plast Reconstr Surg.

[13] Melissinos EG, Parks DH (1989). Post trauma reconstruction with free tissue transfer: Analysis of 442 consecutive cases. J Trauma.

[14] Benacquista T, Kasabian AK, Karp NS (1996). The fate of lower extremities with failed free flaps. Plast Reconstr Surg.

[15] Ponten B (1981). The fasciocutaneous flap: Its use in soft tissue defects of the lower leg. Br J Plast Surg.

[16] Mooney JF, DeFranzo A, Marks MW (1998). Use of cross-extremity flaps stabilized with external fixation in severe pediatric foot and ankle trauma: an alternative to free tissue transfer. J Pediatr Orthop.

[17] Wells MD, Bowen CV, Manktelow RT, Graham J, Boyd JB (1996). Lower extremity free flaps: A review. Can J Surg.

[18] Serafin D, Georgiade N, Smith DH (1977). Comparison of free flaps with pedicled flaps for coverage of defects of the leg or foot. Plast Reconstr Surg.

[19] Morris AM, Buchan AC (1978). The place of the cross-leg flap in reconstructive surgery of the lower leg and foot: A review of 165 cases. Br J Plast Surg.

[20] Hallock GG (2000). Impact of the successful flap but failed reconstruction on the true rate of success in free-tissue transfers. J Reconstr Microsurg.

[21] Hodgkinson DJ, Irons GB (1980). Newer applications of the cross-leg flap. Ann Plast Surg.

[22] Landra AP (1982). Salvage of a seriously injured lower limb with a musculo-cutaneous cross-leg flap. Br J Plast Surg.

[23] Hudson DA, Millar K (1992). The cross-leg flap: still a useful flap in children. Br J Plast Surg.

[24] Long CD, Granick MS, Solomon MP (1993). The cross-leg flap revisited. Ann Plast Surg.

